# Proteins that mediate protein aggregation and cytotoxicity distinguish Alzheimer's hippocampus from normal controls

**DOI:** 10.1111/acel.12501

**Published:** 2016-07-23

**Authors:** Srinivas Ayyadevara, Meenakshisundaram Balasubramaniam, Paul A. Parcon, Steven W. Barger, W. Sue T. Griffin, Ramani Alla, Alan J. Tackett, Samuel G. Mackintosh, Emanuel Petricoin, Weidong Zhou, Robert J. Shmookler Reis

**Affiliations:** ^1^ McClellan Veterans Medical CenterCentral Arkansas Veterans Healthcare ServiceLittle RockAR72205USA; ^2^ Department of GeriatricsUniversity of Arkansas for Medical SciencesLittle RockAR72205USA; ^3^ BioInformatics ProgramUniversity of Arkansas for Medical Sciences and University of Arkansas at Little RockLittle RockAR72205USA; ^4^ Department of Biochemistry & Molecular BiologyUniversity of Arkansas for Medical SciencesLittle RockAR72205USA; ^5^ Center for Applied Proteomics and Molecular MedicineGeorge Mason UniversityManassasVA20110USA

**Keywords:** Abeta(1‐42), acetylation (protein), aggregation (protein), Alzheimer (Disease), beta amyloid, *C. elegans*, microtubule‐associated protein tau, neurodegeneration, neurotoxicity, oxidation (protein), phosphorylation (protein), proteomics

## Abstract

Neurodegenerative diseases are distinguished by characteristic protein aggregates initiated by disease‐specific ‘seed’ proteins; however, roles of other co‐aggregated proteins remain largely unexplored. Compact hippocampal aggregates were purified from Alzheimer's and control‐subject pools using magnetic‐bead immunoaffinity pulldowns. Their components were fractionated by electrophoretic mobility and analyzed by high‐resolution proteomics. Although total detergent‐insoluble aggregates from Alzheimer's and controls had similar protein content, within the fractions isolated by tau or Aβ_1–42_ pulldown, the protein constituents of Alzheimer‐derived aggregates were more abundant, diverse, and post‐translationally modified than those from controls. Tau‐ and Aβ‐containing aggregates were distinguished by multiple components, and yet shared >90% of their protein constituents, implying similar accretion mechanisms. Alzheimer‐specific protein enrichment in tau‐containing aggregates was corroborated for individuals by three analyses. Five proteins inferred to co‐aggregate with tau were confirmed by precise *in situ* methods, including proximity ligation amplification that requires co‐localization within 40 nm. Nematode orthologs of 21 proteins, which showed Alzheimer‐specific enrichment in tau‐containing aggregates, were assessed for aggregation‐promoting roles in *C. elegans* by RNA‐interference ‘knockdown’. Fifteen knockdowns (71%) rescued paralysis of worms expressing muscle Aβ, and 12 (57%) rescued chemotaxis disrupted by neuronal Aβ expression. Proteins identified in compact human aggregates, bound by antibody to total tau, were thus shown to play causal roles in aggregation based on nematode models triggered by Aβ_1–42_. These observations imply shared mechanisms driving both types of aggregation, and/or aggregate‐mediated cross‐talk between tau and Aβ. Knowledge of protein components that promote protein accrual in diverse aggregate types implicates common mechanisms and identifies novel targets for drug intervention.

## Introduction

Protein aggregation has long been recognized as a common feature of most or all age‐dependent neurodegenerative diseases, and yet very little is known about which features of aggregating proteins contribute to their accrual or their neurotoxicity. Intriguing clues have come from genetic mutations that cause such diseases to recur sporadically in pedigrees, even though familial cases typically comprise only a small fraction of the total (Lio *et al*., 2003; Cardenas *et al*., 2012; Abdel‐Salam, 2014; Barmada, 2015; Heneka *et al*., 2015). Those mutations may render a single ‘seed’ protein susceptible to aggregation in whichever brain regions it is chiefly expressed. Examples include genetic expansion of polyglutamine (polyQ) arrays in the huntingtin protein (Huntington's disease, HD) as well as in ataxins and similar Q‐rich proteins (spinocerebellar ataxias); mutations to SOD1 that favor its aggregation (amyotrophic lateral sclerosis, ALS); mutations in α‐synuclein that cause Parkinson's disease (PD); mutations in A4/APP or presenilins that favor production of amyloid β peptide, Aβ_1‐42_ (Alzheimer's disease, AD); and mutated forms of tau that are predisposed to form neurofibrillary tangles (certain familial AD pedigrees, other tauopathies) (Jellinger, 2012). Some neuropathic mutations, however, may interfere more broadly with the normal clearance of diverse misfolded and aggregated proteins. Proteins harboring those mutations include ubiquilin‐1, leading to presenilin aggregation (mutated in some familial AD) (Viswanathan *et al*., 2011); ubiquilin‐2 which also targets ubiquitinylated proteins to proteasomes (mutated in ALS) (Zhang *et al*., 2014); and parkin, an E3 ubiquitin ligase required for mono‐ubiquitin addition to specific protein targets (mutated in PD) (Roy *et al*., 2015). Other predisposing factors can markedly elevate the risk of specific neurological diseases, for example, brain trauma, epilepsy, hypertension, obesity, type 2 diabetes, and exposure to toxic chemicals (in AD, PD) (Vosler *et al*., 2008; Zigman, 2013). Age is a major risk factor for almost all neuropathies, as well as an important determinant of disease progression (Brehme *et al*., 2014; Fjell *et al*., 2014).

Factors that confer risk for progressive neurodegenerative diseases, although diverse, are all consistent with a simple unifying hypothesis: Protein aggregation leads to neural damage and is exacerbated by aging and other pro‐inflammatory triggers (David *et al*., 2010; Dillin & Cohen, 2011). Aggregates are neurotoxic (or more generally cytotoxic) in at least some forms (Bucciantini *et al*., 2002); it remains unresolved whether the greater threat is posed by relatively soluble and diffuse aggregates (typically oligomeric) or insoluble, compact conglomerates (Tai *et al*., 2013). Protein aggregates have been shown to form even during normal aging of nematodes (David *et al*., 2010; Ayyadevara *et al*., 2014). Postsynthetic modifications such as hyperphosphorylation (Rudrabhatla *et al*., 2011) and oxidation (Boyd‐Kimball *et al*., 2006) strongly promote aggregation and may also accrue with age.

We recently characterized protein aggregates formed in a *C. elegans* model of HD, expressing Q40::YFP (a 40‐glutamine tract fused in‐frame to yellow fluorescent protein) in body‐wall muscle. We purified co‐aggregating proteins by YFP‐antibody affinity and analyzed them by high‐resolution proteomics (Ayyadevara *et al*., 2014). We demonstrated in this model that several proteins identified in compact Q40‐containing foci promote aggregation by disrupting proteasomal function, and knockdown of their expression markedly reduced the extent of aggregation in the same HD model and in two *C. elegans* lines expressing a human Aβ_1–42_ transgene (Ayyadevara *et al*., 2014).

We now extend this experimental approach to affected hippocampal tissue from subjects with dementia and histopathology typical of AD, and we compare them to the same regions from normal controls spanning the same age range. The hippocampus, a cortical structure implicated in memory formation, is especially vulnerable in AD. We find higher levels in AD of hippocampal aggregates that bind antibody against Aβ_1–42_ or tau; these immuno‐purified conglomerates contain many proteins that differ markedly and reproducibly in quantity and post‐translational modifications between AD and normal controls. Intriguingly, we find roughly twofold increases in oxidation, phosphorylation, and acetylation of specific proteins enriched in AD aggregates. Selected results were confirmed in additional proteomic analyses, targeted knockdowns, and imaging of proximity ligation products.

## Results

### Proteomic analysis of pure aggregates from Alzheimer and control hippocampus

**Figure 1:**
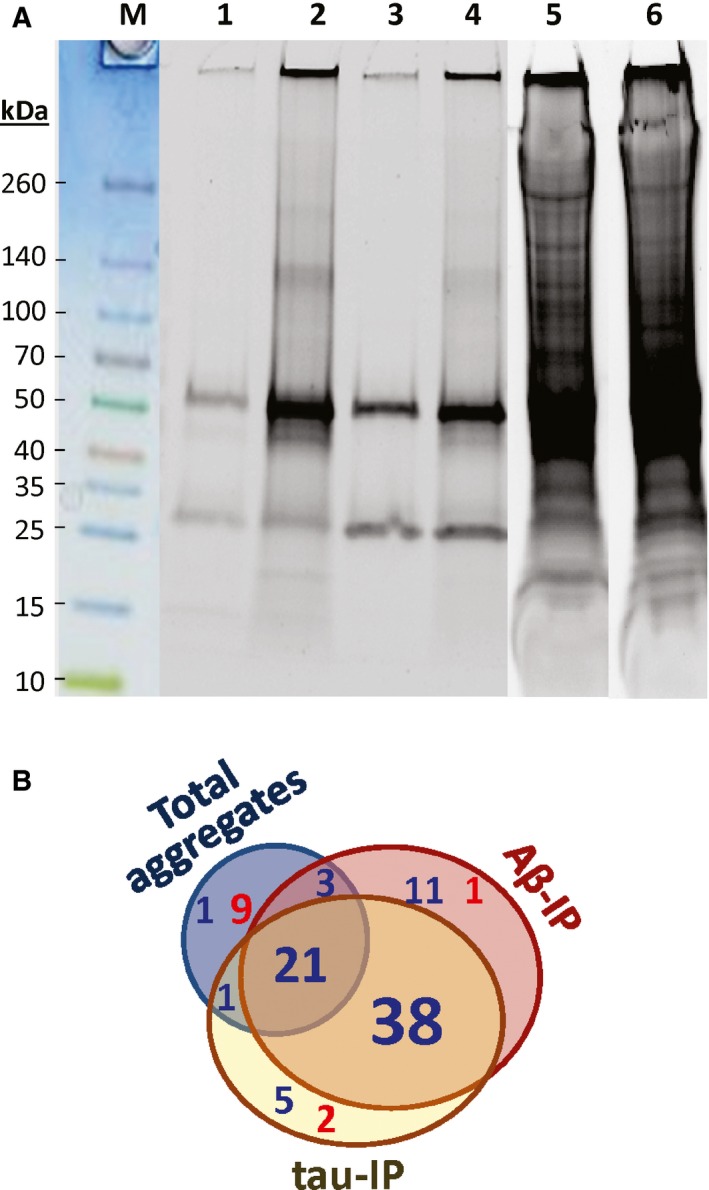
Proteins in detergent‐insoluble AD‐derived aggregates are more abundant and diverse than those from controls. (A) Aggregates insoluble in 1% sarcosyl, isolated from hippocampi of normal controls (3 subjects pooled, lanes 1, 3, and 5) or AD (pool of 3, lanes 2, 4, 6). Lanes 1 and 2: pulldown with antibody to Aβ_1–42_; lanes 3 and 4: pulldown with antibody to tau; lanes 5 and 6: total large insoluble aggregates. (B) Venn diagram of proteins found to be significantly enriched (blue font) or depleted (red) in aggregates from AD relative to controls. More than half of the proteins listed in Table 1 (59/100) were significantly more abundant in both Aβ and tau pulldowns from AD than from controls.

We first isolated and compared compact‐aggregate fractions from pools of caudal hippocampus obtained from AD vs. normal controls (NC). After differential centrifugation to remove debris and organelles, tissue homogenates were gently mixed with magnetic beads (Dynabeads^™^) coated with antibody to either Aβ_1–42_ or tau. Complexes containing those proteins were bound, rinsed thoroughly, and recovered by elution from the beads. The two immuno‐adsorbed subfractions as well as total large aggregates (prior to affinity isolation) were each partitioned by solubility in 1% sarcosyl to separate ‘compact’ (sarcosyl‐insoluble) aggregates from those that dissolved in this strong ionic detergent, in the presence of 0.3‐m β‐mercaptoethanol as reducing agent (Ayyadevara *et al*., 2014). A typical 1‐D acrylamide gel is shown in Fig. 1A for sarcosyl‐insoluble fractions. *Most, but not all, Aβ‐ and tau‐associated proteins are more abundant in compact aggregates derived from AD samples (lanes 2 and 4) than in those derived from NC samples* (lanes 1 and 3), whereas total signal for large aggregates did not differ in overall intensity between groups (compare lanes 5 and 6). Note, however, that the band at ~30 kDa did not differ appreciably in intensity between AD and NC, providing a sort of fortuitous, unidentified negative control for bands that did alter.

Proteomic analyses of insoluble fractions were conducted as detailed in Experimental procedures. For each fraction, corresponding to lanes 1–6 of Fig. 1A, proteins were separated by preparative gel electrophoresis. Gels were sliced, and the slices (including insoluble material at the top) were robotically excised for trypsin digestion prior to peptide analysis by mass spectroscopy (LC‐MS/MS). No insoluble material was detected after digestion.

Positively identified proteins and their modifications are listed in Table 1. Data columns 1–6, corresponding to lanes 1–6 of Fig. 1, show actual spectral counts, which are proportional to relative protein abundances after adjustment for protein size (Byrum *et al*., 2013). For Aβ immuno‐pulldown (IP), 134 proteins differed significantly (at chi‐squared *P *<* *0.05) between AD and NC, far exceeding the 15 expected by chance (=5% of ~300 proteins with sufficient counts to reach significance; see Experimental procedures for details, and Table 1, for examples). In tau‐IP, 121 proteins differed between AD and NC (at *P *<* *0.05) vs. 11 expected by chance. For total sarcosyl‐insoluble aggregates, which are much more abundant overall, 115 proteins altered significantly whereas 44 would be expected at random.

**Table 1:**
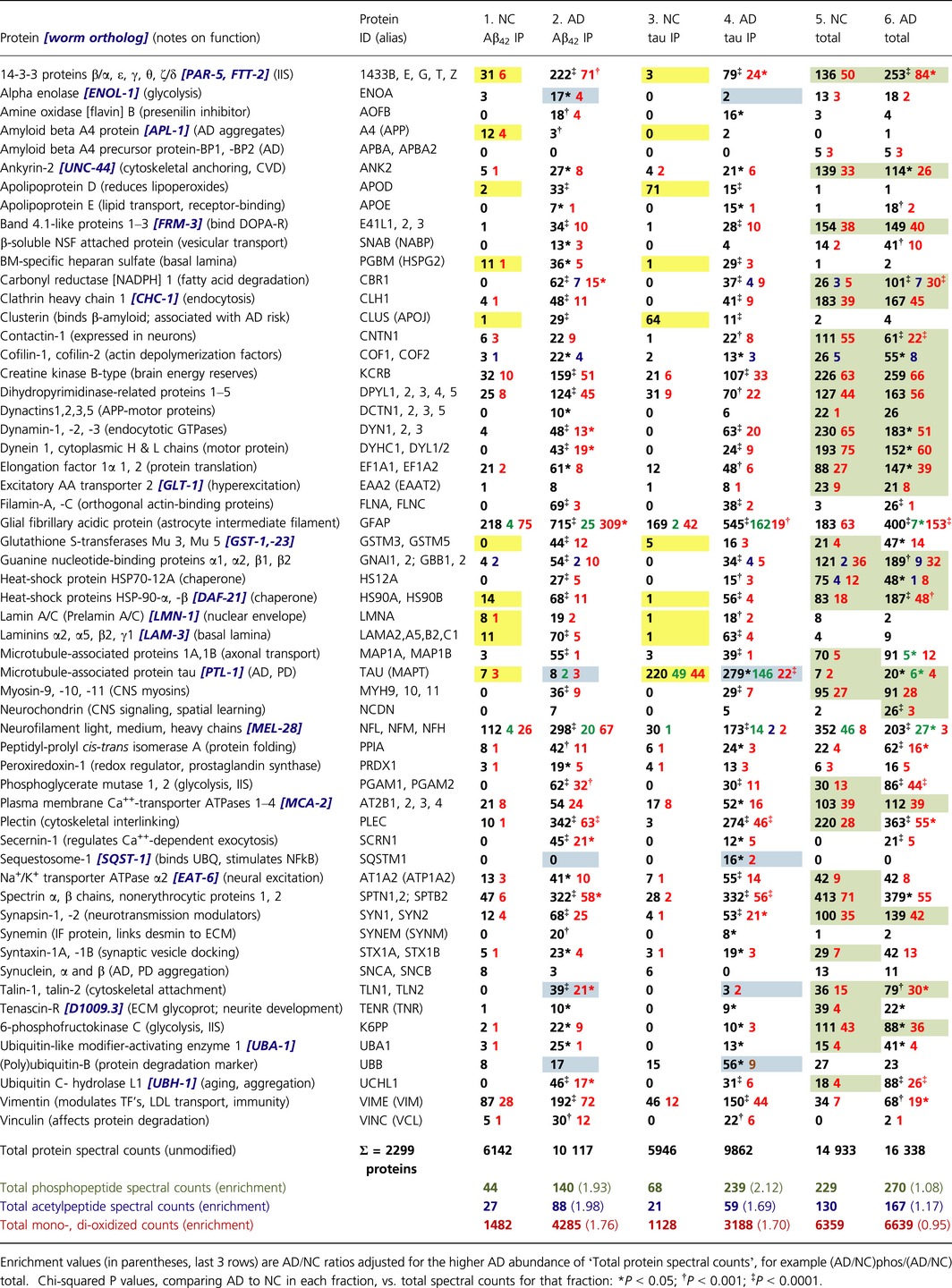
Relative protein abundances in immuno‐purified (IP) vs. total sarcosyl‐insoluble aggregates from AD vs. NC hippocampus. Spectral counts are shown for insoluble fractions: totals in black, oxidized peptides red, phosphorylations green, and acetylations blue

Ten proteins (yellow highlighting in Table 1) clearly distinguish between Aβ_1–42_‐ and tau‐affinity IP in the control samples, some by >30‐fold (tau, apolipoprotein D, and clusterin). The two IPs from AD tissue likewise differ markedly from one another in the abundance of 5 proteins (α‐enolase, sequestosome‐1, tau, and talins 1 and 2), highlighted in blue. Such striking differences demonstrate that distinct aggregate populations were isolated by the two antibodies.

Nevertheless, these data also provide evidence of extensive overlap between proteins co‐aggregating with Aβ_1–42_ vs. tau. Of the 100 proteins listed in Table 1 were significantly more abundant (at χ^2^ *P *<* *0.05) in aggregates from AD hippocampus than from NC, after *both* Aβ‐ and tau‐affinity IP (Fig. 1B). These include many proteins associated with *axonal transport* (tau, dynactins, dynamins, dynein 1, filamins, GFAP, cofilins, microtubule‐associated proteins MAP1A & B, and neurofilament chains NFL, NFM, & NFH), *neurotransmission* (band 4.1‐like proteins, β‐SNAB, synapsins, synemin, and syntaxins), *other neuronal processes* (amine oxidase B, contactin 1, excitatory AA transporter 2, Na+/K+ transporter ATPase α2, tenascin receptor), *redox control* (peroxiredoxin 1, carbonyl reductase, and Mu‐class GSTs), *signal transduction* (14‐3‐3 proteins, elongation factor 1α, G_α, β_ proteins, 6‐phosphofructokinase C), and *proteostasis* (heat‐shock proteins, MAP1 complex, peptidyl‐prolyl isomerase, sequestosome‐1, UBA‐1, polyubiquitin B, ubiquitin C‐terminal hydroxylase, vinculin). In contrast to those AD‐associated increases, α and β synucleins, characteristic of Parkinson inclusions (Park *et al*., 2002), were *depleted* in tau‐ and Aβ‐IP aggregates derived from AD hippocampus.

All of the above AD‐NC changes were peculiar to the tau‐ and Aβ‐pulldown fractions and were severely blunted in total aggregates, isolated by sarcosyl insolubility without IP. Of the 59 proteins with higher representation in AD‐derived tau‐ and Aβ‐IP aggregates, 21 were more modestly (but significantly) enriched with AD in *total* large aggregates (data columns 5 and 6), whereas 9 were depleted in AD samples. Total aggregate protein far exceeds the sum of IP fractions, as is apparent in Fig. 1A (note lighter exposure for lanes 5 and 6), Table 1, and Supplemental Spreadsheets, reflecting the age‐dependent accrual of protein aggregates even without a pathogenic ‘seed’ protein (David *et al*., 2010; Ayyadevara *et al*., 2014). However, pronounced AD/NC bias was rare in total aggregates, where it was seen only for apolipoprotein E, neurochondrin, secernin‐1, and tau, with an intermediate shift also evident for filamins A and C, β‐SNAB, and carbonyl reductase.

Peptides modified by oxidation, phosphorylation, or acetylation were identified and, with few exceptions, were substantially enriched in Aβ_1–42_‐ and tau‐affinity pulldowns from AD (Table 1, colored numbers, summarized in the bottom rows and in Fig. S1; see also (Boyd‐Kimball *et al*., 2006)). Oxidations, the most common modifications (typically 25–44% of total hits), increased nearly threefold in AD relative to normal controls (>1.7‐fold even after adjusting for 66% more aggregates observed in AD), in both Aβ_1–42_‐ and tau‐affinity aggregates (for each IP, *P *=* *0.006 by 2‐tailed paired *t‐*tests; *P *<* *10^−6^ by Fisher exact tests) but not in *total aggregates* from AD subjects (AD ≈ NC). Oxidation of AD‐aggregate proteins was 31% more frequent in Aβ_1–42_‐IP aggregates than in tau‐IP aggregates (2‐tailed paired *t‐*test *P *<* *0.008), as might be expected from the predominantly extracellular sites of Aβ plaque. The extent of oxidation thus varies among aggregate proteins, reflecting variation in susceptibility and also differs between aggregate classes. For example, oxidized sites were found in 22–23% of all neurofilament‐chain peptides from Aβ‐IP aggregates but were seen in only 1–2% of the same peptides from either total or tau‐IP aggregates (chi‐squared *P *<* *10^−30^ for each difference). In contrast, elongation factor 1α is far more oxidized in total aggregates (27–31%) than in Aβ‐ or tau‐IP aggregates (0–13%). Oxidized proteins are thought to be more vulnerable to aggregation and were found to be elevated in a *C. elegans* model of AD (Boyd‐Kimball *et al*., 2006) and in hippocampi of AD and aging Down's patients (Butterfield *et al*., 2014; Di Domenico *et al*., 2014). However, this report is the first (to our knowledge) to identify and quantify proteins and modifications from specific affinity‐purified aggregates, and the first to show unequal distribution of oxidized proteins among aggregate types, which must reflect differing properties of those classes (see Discussion).

Phosphorylations were seen in 285 proteins, 12% of those detected. Summing over all aggregate proteins, phosphorylations were 3.2‐ and 3.5‐fold more frequent within Aβ‐ and tau‐containing aggregates, respectively, from AD vs. NC samples (1.9‐ and 2.1‐fold after adjustment for aggregate quantities) but were scarcely elevated in total aggregates (*P *<* *1E–7 by Fisher's exact tests). Although tau‐IP aggregates were purified with antibody to *native* tau, the increase of tau peptides in AD samples was largely due to higher abundance of tau phosphopeptides in AD; the fraction of phosphorylated tau peptides was >52% for AD, vs. 22% for NC (*P *<* *10^−11^). Among other proteins recovered from aggregates, phosphorylated peptides comprised 0–8% of total peptides (e.g., 3 neurofilament chains and GFAP), averaging twofold to threefold higher levels in AD than NC samples. Acetylations were only half as frequent as phosphorylations in these aggregates, but were likewise enriched in samples derived from AD relative to NC: 3.3‐fold after Aβ‐IP, 2.8‐fold after tau‐IP (2‐ and 1.7‐fold with adjustment for aggregate quantities; each *P *<* *0.005), but <1.3‐fold in total large aggregates (Table 1, last 4 rows). These data could imply that acetylation, phosphorylation, and oxidation all promote aggregation of at least some proteins. Other explanations, such as increased susceptibility to modification of proteins in specific aggregates, cannot be excluded by the data presented. In view of the consistent lack of AD enrichment for similar modifications in total aggregate proteins, however, a purely artefactual explanation appears unlikely.

### Meta‐analysis of aggregate proteins that are differentially represented in AD vs. controls

We analyzed the 89 most differentially represented proteins for functional‐annotation term enrichment using GO/pathway analysis (DAVID ver. 6.7, https://david.ncifcrf.gov/; *Nature Protocols* 2009; 4: 44). The top GO and KEGG terms identified were as follows:

**Table:** 

Term	*N*	Enrichment	FDR (Benj.)
Cytoskeleton	39	4.9	4.4E–16
Acetylation	45	4.1	6.0E–16
Cytoplasm	49	3.5	1.2E–15
Phosphoprotein	67	2.2	1.5E–14
Neural projection	20	10.1	3.8E–12
14‐3‐3 proteins	6	216	5.1E–11
Actin binding	17	9.4	1.8E–09
P‐ser interaction	6	202	1.1E–08
Intracellular transport	19	5.4	5.1E–06
GTP binding	7	24.8	1.2E–05
ATPase, G type	5	69.4	3.9E–05
Disease mutation	22	3.3	4.1E–05
Microtubule	9	9.3	1.3E–04
Neurodegeneration	5	14.8	4.4E–03

These highly significant enrichments corroborate processes that were also suggested by the differential protein abundances of Table 1. Specifically, acetylation and phosphorylation of proteins (and proteins that recognize other phosphorylated proteins) are important to aggregation, as are pathologic genetic mutations. The cell structures most closely associated with aggregate components are the cytoskeleton, cytoplasm, axons, and dendrites, with particular involvement of microtubules, neurofilaments and actin‐binding proteins. Related processes include neurodegeneration and heritable diseases, as well as intracellular transport, a variety of ATPases, and GTP binding proteins.

### Proteomic analyses of individual cortical samples

The above proteomic studies compared pooled hippocampal tissue from 3 AD cases to a similar pool from 3 normal controls. To assess within‐ and between‐group variation in the protein composition of aggregates, we compared proteomic data for tau‐IP samples from caudal hippocampus, analyzed individually for 4 AD vs. 4 age‐matched control (AMC) subjects, 61–85 years of age (Table S1). This comparison was not intended to precisely replicate that of pooled samples, which had been fractionated by gel electrophoresis, as individual samples were not similarly prefractionated—an economy that reduced the yield of identified proteins. Nevertheless, many proteins that were most differentially abundant in tau‐IP aggregates from AD vs. control pools also differed significantly between AD vs. AMC groups when individual aggregate samples were analyzed. In this analysis, 25 proteins were significantly enriched in aggregates from AD tissue, at *t*‐test *P *<* *0.05. Table 2 lists 20 AD‐enriched proteins from this comparison, with AD/AMC ratios >1.5, and their *P* values (in 1‐tailed heteroscedastic *t*‐tests, appropriate to 1‐way comparisons for samples of this size) (“tau‐IP 1” columns). Table 2 also includes data from a technical repeat comparing 4 AD to 4 NC samples using a different LC‐MS platform and protocol (“tau‐IP 2” columns). The correlation coefficient between technical replicates for AD/AMC ratios of all 25 tau‐IP proteins was *R *=* *0.80 (*P *<* *1E–6), or 0.83 (*P *<* *4E–5) considering only 11 proteins with *P *≤* *0.08. The last column, tau pools, shows corresponding hits for pooled samples (Table 1).

**Table 2:**
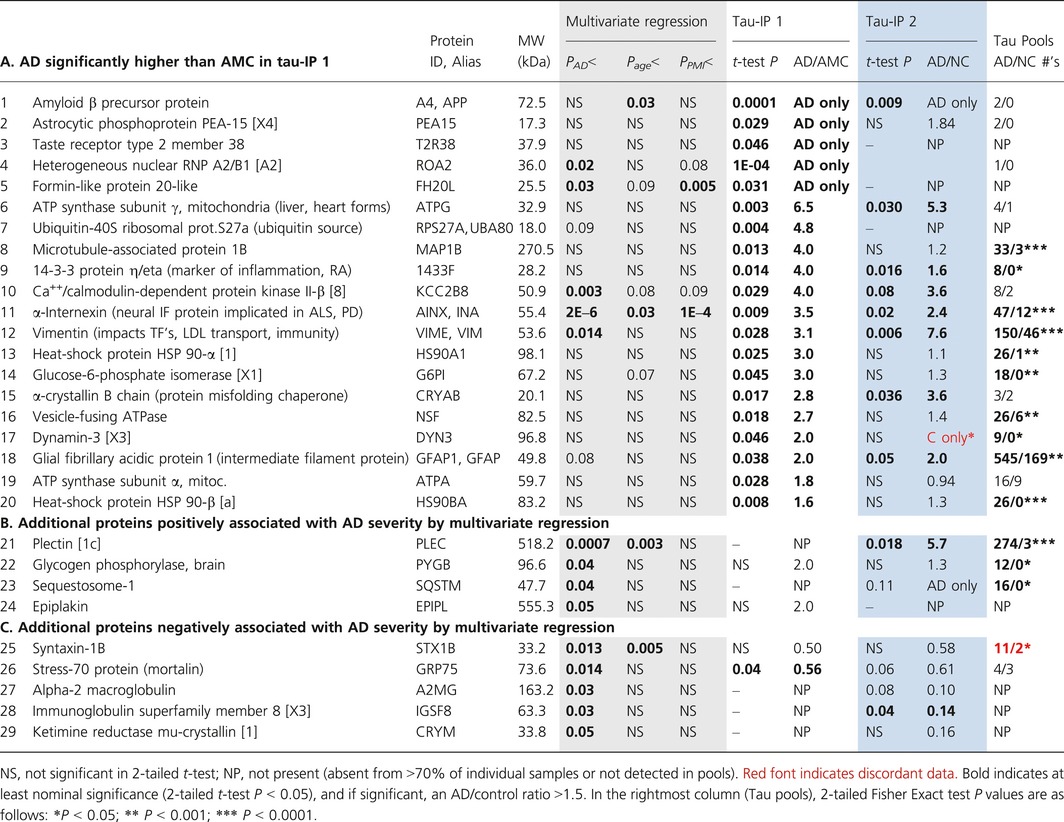
Proteins in tau immuno‐purified hippocampus aggregates: Differential abundance for AD vs. AMC individuals

In the primary analysis, 235 ± 50 (mean ± SD) proteins were identified per individual in tau‐IP aggregates, and a similar number (264 proteins) were present in enough individuals to have the potential to indicate a significant shift between AD and AMC. Of these, 12–13 proteins are expected to appear differential by chance alone at α ≤ 0.05, whereas 25 were enriched in AD aggregates by this criterion. Because up to half of the candidate proteins might be false positives, we limited Table 2 to 20 proteins showing pronounced, replicable shifts. Proteins were omitted due to either low AD/AMC ratios (1.2 for tubulin chains α‐4A and β‐4B) or lack of support from other comparisons (elongation factor Tu, ADP/ATP translocase 2, and 2‐oxoglutarate dehydrogenase E1). Proteins *less* abundant in AD than AMC aggregates (glutamate dehydrogenase, aspartate aminotransferase, stress‐70/mortalin, and keratins I‐13 and I‐14) were excluded by our predetermined focus on AD increases.

Proteins significantly and reproducibly enriched in tau‐IP aggregates from AD relative to AMC (Table 2, “tau‐IP 1”) include amyloid precursor protein (APP or A4), astrocytic phosphoprotein PEA‐15, formin‐like protein 20‐like, heterogeneous nuclear ribonucleoprotein [hnRNP] A2/B1, and taste receptor 2‐38 (all found only in AD); ATP synthase (α and γ subunits), calcium/calmodulin‐dependent protein kinase II‐β, ubiquitin‐40S ribosomal protein, 14‐3‐3 protein η (eta), microtubule‐associated protein 1B, vimentin, α‐internexin, glucose‐6‐phosphate isomerase, HSP90‐α and HSP90‐β, α‐crystallin B chain, vesicle‐fusing ATPase, dynamin‐3, and GFAP‐1.

We extended this comparison to a larger sample (8 AD and 7 NC) spanning a wider age range (46–92 years) and analyzed 73 candidate proteins by multivariate regression against each of the potentially relevant parameters (Table 2, multivariate regression columns). Effects of gender and race were not tested due to insufficient sample size for one or more groups; likewise, ApoE genotype (a strong risk factor for AD) was not included in regressions due to its close coupling to AD in our sample (see Table S1). Tau‐IP levels of 3 proteins showed nominally significant associations with postmortem interval (PMI), and levels of 4 proteins covaried with age (close to the frequencies expected by chance). In contrast, AD severity based on immunohistochemical features (Braak *et al*., 2006) was significantly linked to aggregate tau‐IP levels of 13 proteins (18%) after adjustment for age and PMI effects—substantially more frequently than expected by chance.

Positive associations with AD severity were found for α‐internexin (*P *<* *2 × 10^−6^), plectin (*P *<* *0.0007), Ca^++^/calmodulin‐dependent protein kinase II‐β (*P *<* *0.003), vimentin (*P *<* *0.014), hnRNP A2/B1 (*P *<* *0.02), formin‐like protein 20‐like (*P *<* *0.03), glycogen phosphorylase B (*P *<* *0.04), sequestosome‐1 (*P *<* *0.04), and epiplakin (*P *<* *0.05). Negative regressions were nominally significant for stress‐70/mortalin (*P *<* *0.014), syntaxin‐1B (*P *<* *0.013), α2‐macroglobulin (*P *<* *0.03), immunoglobulin superfamily member 8 (*P *<* *0.03), and ketamine reductase mu‐crystallin 1 (*P *<* *0.05).

### *In situ* confirmation of protein–protein associations and their enrichment in AD aggregates

**Figure 2:**
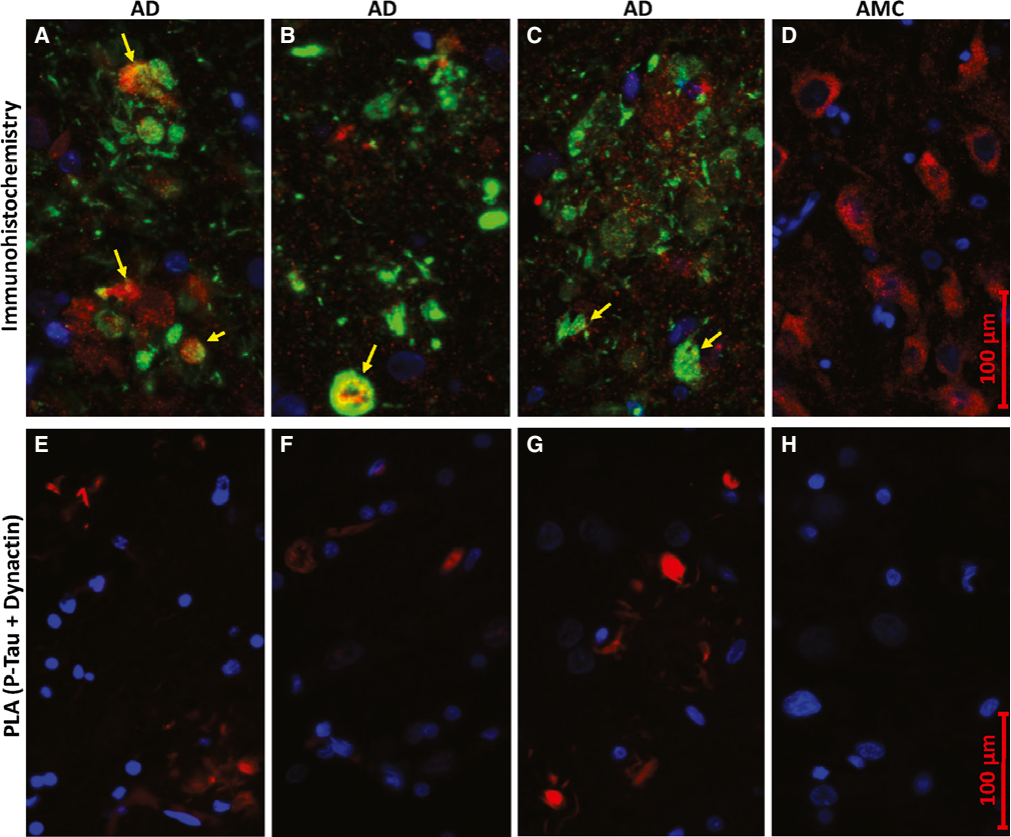
P‐tau coincides with dynactin *in situ*. Images show hippocampus sections from Alzheimer disease (A–C, E–G) or age‐matched controls (D, H). (A–D) Conventional immunohistochemistry, visualizing proteins with fluor‐tagged secondary antibodies, after incubation with antibodies to P‐tau (Ser202, Thr205; green) and dynactin/p50 (red); nuclei (blue) were stained with DAPI. Light green and yellow (arrows) indicate superposition of red and green fluors. (E–H) Proximity ligation amplification (PLA); red product indicates close proximity (<40 nm separation) of P‐tau (Ser202, Thr205) to dynactin/p50.

We used antibodies specific to several of the proteins identified in tau‐IP aggregates to assess whether colocalization by fluorescence microscopy would support or refute the associations implied by our proteomic data. Representative immunostained images are shown in Fig. 2 (upper panels) wherein light green and yellow (e.g., arrows) imply coincidence between phospho‐tau (P‐tau) imaged as green, and dynactin shown as red. P‐tau [Ser202, Thr205] was detected in most AD but none of the AMC neurons, consistent with our proteomic data (threefold more P‐tau peptides in AD, summed over *all* phosphorylation sites; Table 1). Dynactins were equally abundant in AD and AMC neuron bodies, again in agreement with proteomic results (Table 1, ‘total’ aggregate columns). These observations imply that a substantial fraction of dynactin colocalizes with some portion of P‐tau (presumably intracellular, as in control neurons of panel D). In the absence of any P‐tau signal for AMC tissue, it is not possible to draw any conclusions regarding P‐tau interaction with dynactin in normal cells.

Proximity ligation amplification (DuoLink PLA, Fig. 2 lower panels) provides a much more stringent test of co‐localization, as rolling‐circle amplification primers coupled to two distinct secondary antibodies can greatly amplify their product (detected as red fluorescence) only if they are situated within 40 nm of each other. PLA results place tighter limits on the coincidence of P‐tau and dynactin signals in AD, noted above, and corroborate the data of Table 1 indicating enrichment of dynactins in tau‐IP aggregates prepared from AD but not AMC hippocampus.

**Figure 3:**
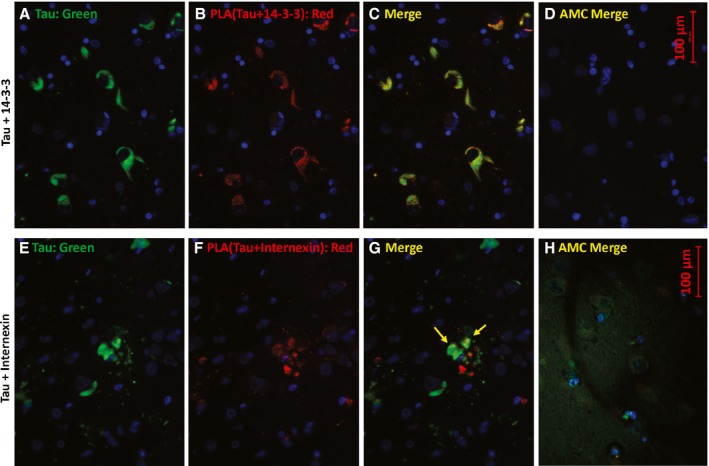
Total tau colocalizes with 14‐3‐3 and a subset of internexin in AD hippocampus. Sections were immunostained for total tau (A, E; green), or by proximity ligation amplification (PLA) for total tau in conjunction with 14‐3‐3 proteins (B; red) or with internexin (F; red). C and G are merged images of A+B and E+F, respectively. D and H are the corresponding merged images for AMC hippocampus. Note that in H, the tau (green) exposure was increased ~10‐fold to illustrate the diffuse distribution of normally phosphorylated tau in control tissue.

**Figure 4:**
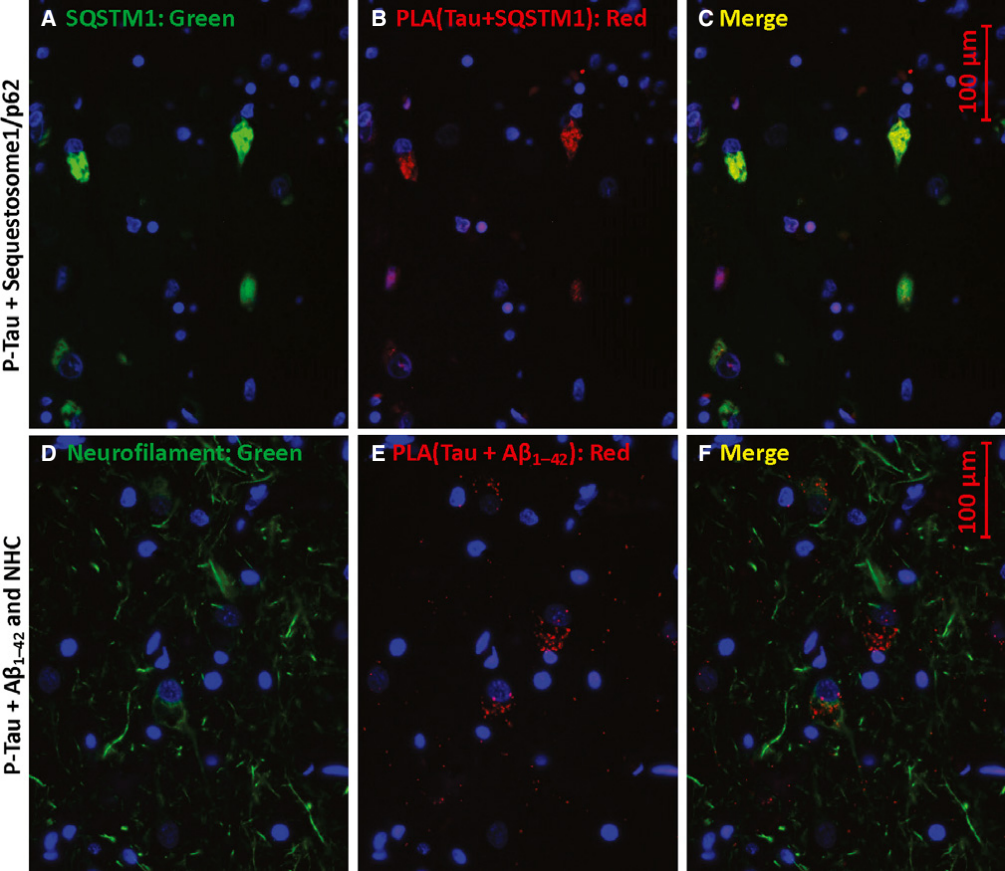
P‐tau proximity to sequestosome‐1 and Aβ_1–42_/APP. Sections were immunostained for sequestosome‐1 (SQSTM1, p62; A) or neurofilament heavy chain (NHC, D) and for PLA product (red) showing P‐tau (Thr205) proximal to SQSTM1 (B) or to Aβ_1–42_ (E). C and D are merged images of A+B and D+E, respectively.

**Figure 5:**
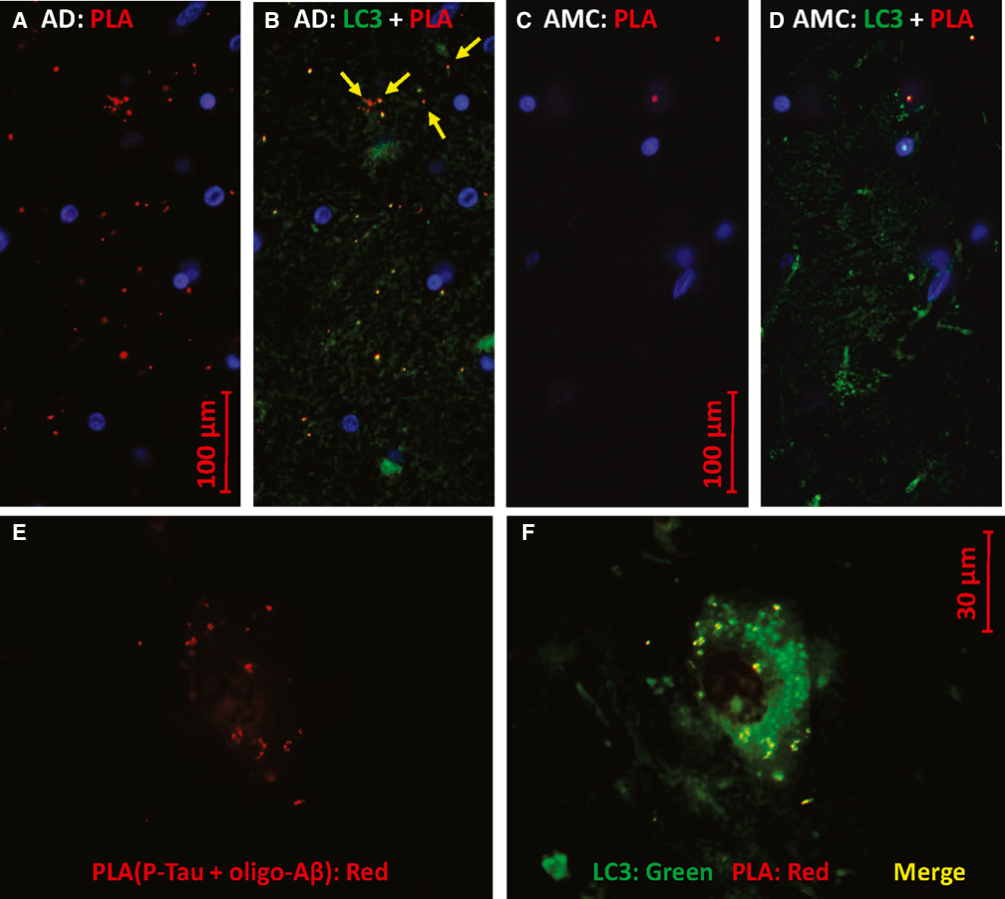
P‐tau co‐localizes with Aβ_1–42_ oligomers. Antibodies to P‐tau [Thr205] and oligomeric Aβ_1–42_ (A–D) produce red PLA product if they are within 40 nm. Counterstaining with antibody to LC3B/ATG8, a marker of autophagosomes (B, D, F), appears as green. In E and F, PLA product (red) indicates P‐tau contiguous to Aβ_1–42_ oligomers. AD hippocampus sections are shown in A, B, E, and F, while C and D show AMC hippocampus.

A total of 5 protein–protein interactions implicated by proteomic tau‐IP results were tested and confirmed by PLA, in some cases coupled with conventional immunofluorescent counterstaining (Figs 2, 3, 4, 5). Consistent and fairly uniform co‐localization of total tau with 14‐3‐3 proteins was observed in AD (Fig. 3A–C) but not AMC hippocampus (Fig. 3D), whereas tau clusters differed widely in the extent of labeling with internexin‐tau PLA product, also seen only in AD (Fig. 3E–H).

Similarly, P‐tau colocalizes with sequestosome‐1/p62 in AD hippocampus (Fig. 4A–C) and with Aβ_1–42_ which appears to be intracellular and located in neurites (Fig. 4D–F) of cells also immunostained for neurofilament heavy chain (NHC, green). The green counterstain in Fig. 5 was antibody to LC3B/ATG8, a microtubule‐associated light‐chain protein with an ubiquitin‐like domain, widely used to mark autophagosomes. Abundant PLA signal, indicating P‐tau contiguous to Aβ_1–42,_ was seen in LC3‐positive puncta of AD hippocampus (panels A, B, E, F) but only rarely in AMC (panels C, D). The antibody for these PLA reactions is specific to Aβ_1–42_ residues 1–4 and does not bind to APP (Youmans *et al*., 2012); it preferentially labels neurotoxic oligomers of Aβ_1–42_ (Tai *et al*., 2013). These data imply that P‐tau complexes with Aβ_1–42_ form inside AD neurons and are sequestered in a subset of autophagosomes. Apparent exceptions (e.g., red features at the top of Fig. 5B) all contain yellow subregions indicating co‐localization.

### Aggregate proteins enriched in AD play functional roles in aggregation and cytotoxicity

We recently reported that several aggregated proteins identified in a *C. elegans* model of Huntington disease (strain AM141, expressing polyglutamine [Q40] fused in‐frame to YFP, yellow fluorescent protein) contribute mechanistically to accumulation of aggregates by obstructing their proteolytic clearance.

**Figure 6:**
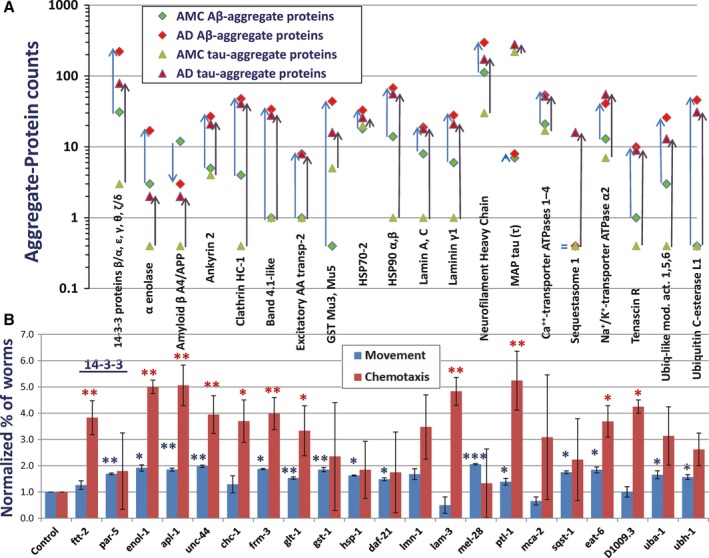
Knockdown of AD‐enriched aggregate proteins rescues aggregation‐induced traits. (A) Proteins shown are overrepresented (in LC‐MS spectral counts) in aggregates isolated from AD relative to NC, except microtubule‐associated protein tau, which is hyperphosphorylated but not more abundant in AD (Table 1). Blue arrows connecting diamond symbols show increased abundance of proteins in tau‐IP aggregates; black arrows connecting triangles show increased abundance in Aβ‐IP aggregates. (B) Of 21 protein types more abundant in AD than in NC aggregates, knockdown of 14 (67%) of their nematode orthologs significantly rescued paralysis of a *C. elegans* strain expressing human Aβ_1‐42_ in muscle (blue bars), or rescued chemotaxis disrupted by neuronal expression of Aβ_1‐42_ (red bars). Significance was assessed by 1‐tailed *t*‐tests (as lower cytotoxicity was predicted) comparing fractions of unparalyzed or chemo‐attracted worms in three independent assays per group (*P <* *0.05; **P <* *0.005; ***P <* *0.0005). Significance by chi‐squared tests *within* assays was *P *<* *5 × 10^−5^ to 10^−16^.

In a similar fashion, we here used *C. elegans* amyloidopathy models to test the effects of RNA‐interference (RNAi) knockdowns targeting 21 candidate genes that encode proteins orthologous to constituents substantially more abundant in insoluble aggregates from AD than AMC hippocampus, when isolated by affinity to tau‐ or Aβ‐specific antibodies (Tables 1 and 2). These orthologs are shown in bold, blue font, in the first column of Table 1. Knockdown results for muscle or neuronal expression of human Aβ_1‐42_ are presented in Fig. 6B, paralleling a diagrammatic representation of aggregate‐specific shifts in protein abundance (Fig. 6A). A strain expressing the human Aβ_1‐42_ transgene in muscle showed extensive paralysis, retaining only ~40% motility at 1.5 d after induction. Paralysis was attenuated by 15 of the knockdowns (71%) with at least nominal significance (vs. 1 expected by chance at *P *<* *0.05; see blue bars, Fig. 6B). The extent of rescue ranged from 28% to 71% (mean ± SD: 53 ± 14%). A second strain, with pan‐neuronal expression of Aβ_1‐42_, lost ~90% of chemotaxis to *n*‐butanol by 2–3 days postinduction. Knockdown of 12 genes (57%) significantly protected from loss of chemotaxis (at *P *<* *0.05; for 6 of those genes, *P *<* *0.005), indicated by red bars in Fig. 6B. The extent of rescue was 30–57% (mean ± SD: 42 ± 9%). In each case, to be as conservative as possible, significance is based on *t*‐test comparisons of biological replicates only (i.e., *N *= the number of repeats). Seven of the 21 tested genes produced significant results in both assays. Although most genes that produced a significant impact did so in only one assay, this is not surprising as AD‐aggregate proteins (and their *C. elegans* orthologs) are likely to be expressed at quite different levels in different cell types.

## Discussion

### Distinct aggregates isolated by Aβ or tau‐IP are both highly enriched for AD‐specific proteins

Aggregates isolated by Aβ IP (thought to reflect initiation or ‘seeding’ by Aβ oligomers) differ markedly with respect to several protein constituents from parallel preparations isolated by tau‐IP (Table 1). This confirms that amyloid plaque aggregates were successfully resolved from neurofibrillary tangles. Considering only aggregates from control hippocampus (yellow highlighting in Table 1), 14‐3‐3 proteins, heparin sulfate, HSP90, lamin A/C, and laminins were detected >10 times as often in Aβ‐IP (75 hits) as in tau‐IP aggregates (7 hits). Conversely, ApoD and clusterin taken together were 45 times *less abundant* (3 vs. 135 hits) when associated with Aβ than when associated with tau. The same points apply to aggregates from AD hippocampus (blue highlighting), but different sets of proteins were most differential between aggregate types: α‐enolase and talins 1 and 2 are >10 times as abundant (56 vs. 5 hits) when associated with Aβ as when linked to tau, while sequestosome‐1 is *only* found in tau aggregates, and polyubiquitin (UBB) is 3.3 times as abundant there.

The ability to distinguish immunologically between aggregates cannot be taken for granted, given that Aβ aggregates contain some tau, and tau‐affinity aggregates (at least from AD tissue) contain a little Aβ [or APP, which may also be recognized by most antibodies to Aβ (Youmans *et al*., 2012)]. Nevertheless, the amount of ‘crossover’ (based on data in Table 1) is relatively small: Tau was identified in Aβ aggregates at only 3% of the level in tau‐affinity aggregates (15 vs. 499 hits), whereas Aβ was also quite scarce in tau tangles compared to Aβ aggregates (2 vs. 15 hits).

It is noteworthy that 90% (90/100) of the proteins comprising these aggregates were enriched (or in a few cases depleted) in AD relative to NC, concordantly for *both* tau‐IP and Aβ‐IP aggregates. Most shifts (74%) were significant in both, even after adjusting for total protein (higher in AD aggregates). *Thus, the ‘aggregate proteome’ is remarkably distinctive for AD and could be useful diagnostically, in addition to shedding light on mechanisms of protein accrual*.

Total aggregates increase with age in normal *C. elegans* (David *et al*., 2010; Ayyadevara *et al*., 2014) and in several tissues of normal mice (Ayyadevara *et al*., 2016 and unpublished data), indicating that protein aggregation is a quite broadly conserved feature of aging. In view of our evidence for common processes underlying the growth of both Aβ and tau aggregates, as discussed above, it is surprising that ‘total aggregates’ (sarcosyl‐insoluble material without IP) differed far less between AD and NC than did either type of neuropathological aggregate. That is, although total aggregates did differ in composition between these two sources (115 proteins differed with chi‐squared *P *<* *0.05 vs. 40 expected by chance), the ratios were far less extreme than those in Aβ and tau aggregates (Table 1). This implies that aggregates are heterogeneous and that those containing either tau or Aβ are more specific to AD than aggregates overall—many of which are likely to reflect age‐dependent proteostasis failure unrelated to the specific pathophysiology of AD (Morimoto & Cuervo, 2014).

### Aggregate proteomics are more informative for AD than total proteomics

AD‐specific proteins comprised a much larger fraction of total proteins in immuno‐isolated aggregates (our data) than in total protein prepared from affected frontal cortex *without* isolation of aggregates (Emory ADRC Brain Bank, https://www.synapse.org/#!Synapse:syn2580853/wiki/). In a proteomic comparison that identified 8027 proteins from a total of 16 pools representing affected tissue from 7 neurological diseases (plus controls), the mean ± SD number of proteins identified per pool was 3508 ± 163. Of those proteins, only 28 (0.8%) and 34 (1.0%) differed with nominal significance (*P *≤* *0.05) between AD and ‘AD/PD’ (PD with some AD traits), respectively, and the common control pools. These results imply that aggregate proteomes are much less complex (comprise fewer proteins) than total tissue proteomes and yet have a far higher fraction of identified proteins that differ significantly between AD and controls. Moreover, although >85% of the proteins identified in aggregates were also identified in the Emory protein set, very few proteins appeared significant in both assays. Of 136 proteins in Tables 1 and 2, only four differed significantly between AD and NC total protein pools in the Emory study: APP, tau, NADH dehydrogenase (NDUFA4), and secernin‐1 (SCRN1). Conversely, proteins that differed significantly between AD/PD and NC in the Emory study included only 6 proteins that also appeared significant in our comparisons: tau, 14‐3‐3γ, HSPβ‐1, peroxiredoxin‐2, required for meiotic nuclear division protein 1 (RMND1), and pyridoxal phosphate phosphatase (PDXP). Even considering all seven neuropathology pools, only 17 proteins were significant in both the Emory study and our own. In contrast, our tables include 15 of the 26 proteins (58%) identified as potentially differential in amyloid plaque isolated by laser‐capture microdissection from two AD cases (Liao *et al*., 2004). The above results are consistent with our hypothesis that neurotoxic aggregates have a protein composition quite distinct from the affected tissues in which they reside.

### Post‐translational modifications contribute to AD‐specific aggregates

Table 1 summarizes proteomic data for oxidations (summing over mono‐, di‐, and trioxidation), phosphorylations and acetylations. These PTMs are elevated 2.8‐fold to 3.5‐fold in AD relative to NC (1.7‐ to 2.1‐fold even after adjustment for increased aggregation in AD), *only in A*β*‐ or tau‐IP aggregates* but *not* in total aggregates. These changes add charged, predominantly nucleophilic sites that may block normal protein interactions, and could destabilize hydrophobic regions otherwise buried in the protein interior. Subsequent misfolding exposes hydrophobic residues that can then coalesce with hydrophobic ‘extrusions’ from other proteins. Some PTMs, in particular oxidations, may arise from elevated ROS levels typical of AD neuroinflammation (Liu *et al*., 2011; Heneka *et al*., 2015). *It is revealing that AD does not affect total aggregates in the same way, implying that the underlying molecular dysfunction does not generalize to types of aggregates that accompany normal aging*. Moreover, total aggregates provide a sort of negative control which serves to exclude most sources of artefactual modification, such as oxidations occurring in lysates during aggregate isolation. While such PTMs might occur or alter after lysis, additional *ad hoc* assumptions are needed to explain how they could distinguish between aggregate types.

In addition to these PTMs, specifically sought during peptide identification under Mascot, we also detected higher levels of polyubiquitin (UBB)—presumably reflecting post‐translational tagging of proteins—in aggregates from AD than from NC. UBB was twice as abundant in AD‐derived aggregates isolated by Aβ‐IP and 3.7× more abundant in AD aggregates isolate by tau‐IP (Table 1). Other approaches imply that ubiquitinated proteins accrue in AD due to compromised proteasomal activity (Cecarini *et al*., 2007); higher prevalence of this deficit in neurons than astrocytes (Orre *et al*., 2013) may contribute to the abundance of neuronal proteins in our results.

### Analyses of individual tau‐IP samples confirm AD‐specific protein enrichment in aggregates

We analyzed tau‐IP aggregates from individual samples of caudal hippocampus in order to estimate the intra‐ and intergroup variance and to evaluate the dependence of aggregate protein levels on age, the extent of AD histopathology, and PMI. We found by multivariate regression that age and PMI affected about as many proteins as would be expected by chance (6 and 8%, respectively, of the total tested), whereas the level of AD pathology was significantly associated with the abundance of 13 proteins (26%) in tau‐IP aggregates. Five of these proteins had also differed significantly between AD and AMC in our initial intergroup comparison, but three others had attained significance only in the second group comparison and/or the pooled‐sample analysis. Half of the proteins (13/26) that were significant in either comparison of individual samples, had also reached significance in the pools, but the remainder were insufficiently abundant. The sensitivity of proteomic detection is inevitably limiting, so that any given comparison will yield many false negatives—proteins that are either not detected or are observed at levels too low to meet statistical thresholds.

### *In situ* data support proteomics results

We used standard immunohistochemical procedures and also PLA, a newer method to sensitively and precisely detect sites of close protein:protein contiguity, to provide independent evidence bearing on several key proteomic observations. Multifluor images suggest heterogeneous aggregate structures specific to AD (Fig. 2A–C), while also supporting co‐localization of P‐tau and dynactin. Proximity ligation amplification (Fig. 2E–H) quite specifically indicated structures in AD hippocampus wherein tau and dynactin lie within 40 nm of each other and thus are likely to interact directly. Similarly, tau or P‐tau was shown to coexist in close proximity to 14‐3‐3 proteins, α‐internexin, sequestosome‐1/p62, and Aβ_1–42_ (including its neurotoxic oligomers (Tai *et al*., 2013)) (Figs 3, 4, 5).

Although amyloid beta peptides Aβ_1–42_ and Aβ_1–40_ are best known as components of extracellular plaque, they are also found within neurons (Youmans *et al*., 2012) where neurofibrillary tangles reside. We consistently identified Aβ_1–42_ or its precursor APP in tau‐IP fractions (Table 2, line 1). In all sections of AD hippocampus examined (but never in AMC), we observed abundant PLA signal for tau in close proximity to Aβ_1–42_ oligomers (Figs 4 and 5) in puncta that counter‐stained for the autophagosome marker LC3B/ATG8 (Fig. 5), consistent with their co‐aggregation in AD neurons. Direct interaction between tau and Aβ in aggregates would have important implications for AD etiology, perhaps rendering moot any debate as to which of these molecules is the primary driver of AD pathology.

The 14‐3‐3 proteins recruit misfolded proteins and associated chaperones to dynein motors for transport to aggresomes (Jia *et al*., 2014). Zeta and eta isoforms of 14‐3‐3 were detected in AD/tau and PD aggregates, respectively (Qureshi *et al*., 2013; Plotegher *et al*., 2014). All five 14‐3‐3 isoforms detected here were enriched in AD aggregates, sevenfold after Aβ_1–42_ IP and 26‐fold after tau‐IP (Tables 1 and 2), while dynein H and L chains were exclusively associated with AD aggregates of both kinds (Table 1). The prominence of aggregate proteins involved in retrograde axonal transport agrees well with the prevalence of retromer‐associated genes in AD genomewide association studies (Vardarajan *et al*., 2012). Dynactins, which are also subunits of the dynein motor complex, are less abundant but also specific to both AD IP fractions (Table 1). α‐Internexin, a neural intermediate filament protein implicated in both AD (Dickson *et al*., 2005) and ALS (Page *et al*., 2011), was consistently enriched in AD tau‐IP aggregates (Table 2). Sequestosome‐1, which helps to recruit misfolded proteins and their aggregates to either proteasomes or autophagosomes (Wooten *et al*., 2006), was highly enriched in tau‐IP aggregates exclusively when derived from AD (Tables 1 and 2). In that context, it is not surprising that tau/Aβ_1–4_ PLA signal also colocalizes with punctate immunostain for LC3B/ATG8, a marker of autophagosome membranes (Fig. 5). While these *in situ* data clearly represent just a small sampling of the protein interactions predicted by co‐isolation of the above proteins in total tau‐IP experiments (Tables 1 and 2), it is encouraging that the results of these quite distinct procedures are consistent with one another and extend prior inference of autophagy impairment in AD as well as other neurodegenerative diseases (Cardenas *et al*., 2012).

### Functional assays imply a nonrandom aggregation process

RNAi knockdowns are functional assays that seek morphologic or behavioral changes arising from disruption of specific proteins and pathways. Knockdown data imply that aggregate components are mechanistically involved in the accrual process, at least in nematodes and very likely also in humans, given the high conservation of pathways across vast evolutionary distances. Thus, they may furnish valuable therapeutic targets for AD and other progressive diseases in which aggregation plays a causal role.

Our data also imply that aggregate growth is not an entirely random process, but rather involves something akin to a pathway: protein accrual occurring in an ordered sequence, either obligatory or preferred. This follows from the remarkable observation that aggregation can be greatly attenuated by any of several interventions. Some RNA interference targets reduce aggregates to the extent that they contribute to them, consistent with roles as structural components of aggregates, whereas others exert effects so potent as to suggest that their disruption must block key steps in a sequential accrual process (Ayyadevara *et al*., 2014). If these latter proteins accrued in a stochastic manner, it should not be possible for combined contributions to exceed 100%. However, relatively large effects of RNAi were observed in *C. elegans* aggregation models after RNAi targeted nematode orthologs of 14‐3‐3 proteins, α‐enolase, amyloid precursor‐like protein, ankyrin‐2, clathrin heavy chain, band 4.1‐like protein, excitatory amino acid transporter‐2, GST‐mu, HSPs 70 and 90, lamin, laminins, tau, neurofilament heavy chains, sequestosome‐1, Na^+^/K^+^‐transporter ATPase α2, tenascin‐R, ubiquitin‐like modifier activators, and ubiquitin C‐terminal esterase L1 (Fig. 6B). These RNAi treatments were tested for efficacy in two nematode models. In the first model, 15 of the 21 RNAi treatments significantly restored the loss of motility due to Aβ expression in body‐wall muscle. The mean ± SD rescue was 53 ± 14% of the untreated deficit (controls fed RNAi vector only), and *the sum of rescue effects was 8 times the maximal impairment!* In the second model, 12 RNAi treatments significantly restored the loss of normal chemotaxis that follows pan‐neuronal Aβ expression. In this case, rescue averaged 42 ± 9% of the untreated deficit, *for a sum of rescue effects exceeding 5 times the maximal deficiency*. These data are inconsistent with models in which aggregate constituents coalesce in a random manner, but instead imply a preferred sequence, that is, an ordered series of accretion steps.

### Roles of aggregate proteins enriched or depleted in AD

AD‐enriched aggregates include proteins associated with heat‐shock and unfolded protein responses, inflammation, and/or innate immunity—categories previously shown to be associated with aging (Finch *et al*., 2010; van Oosten‐Hawle & Morimoto, 2014). Examples include ApoE, complement C3, chaperones (e.g., HSP70.2), plectin, ATP‐dependent RNA helicases, BM‐specific heparin sulfate, filamins A and C, lamin A/C, laminin, talin, tenascins, and vinculin.

Many of the proteins that were here characterized as differentially enriched or depleted in aggregates from AD, relative to NC tissue, have been previously implicated in neurodegenerative diseases and/or protein aggregation. For example, ubiquitin carboxy‐terminal hydrolase L1 (which hydrolyzes ubiquitin added to C‐termini of proteins) is mutated in strict association with PD in one pedigree, and a common polymorphism is associated with PD risk (Leroy *et al*., 1998). Moreover, it has also been implicated in Aβ neurotoxicity in AD (Gong *et al*., 2006). Carbonyl reductase is elevated in hippocampi of 3xTg‐AD mice, which have markedly elevated levels of protein carbonylation (Shen *et al*., 2015). Clusterin (ApoJ) polymorphism is associated with AD (Lambert *et al*., 2009), and its circulating levels predict AD progression (Thambisetty *et al*., 2010). Other apolipoproteins are also strongly linked to AD risk: ApoD catalyzes reduction of peroxidized lipids and its levels increase with age and AD, while ApoE has allele‐specific effects on Aβ levels and AD risk (Tai *et al*., 2013; Dassati *et al*., 2014).

We recently showed that neuronal levels of the motor protein dynactin are affected by age, AD, and ApoE genotype (Aboud *et al*., 2015). Dynactin activates dynein chains to couple organelles to microtubules for fast axonal retrograde transport (Waterman‐Storer *et al*., 1997). Suppression of dynein impairs clearance of α‐synuclein aggregates via the autophagosome/lysosome pathway (Li *et al*., 2013). Excitatory amino acid transporter 2 deficiency has been implicated in AD, HD, and ALS‐PD complex (Yi & Hazell, 2006). Filamin C mutations are associated with CVD and with protein aggregation in cardiac cells (Kley *et al*., 2013). Astrocytic GFAP expression is associated with aging and AD (Middeldorp & Hol, 2011); dominant GFAP mutations in Alexander disease cause protein aggregates that contain GFAP, vimentin, plectin, ubiquitin, and small chaperones such as α‐crystallin (Hagemann *et al*., 2009). The 14‐3‐3 proteins, expressed ubiquitously but most abundant in brain, bind as dimers to >200 known target proteins. They were first identified in Lewy bodies of PD, where they are thought to bind α‐synuclein (Plotegher *et al*., 2014). However, they also bind tau in AD neurofibrillary tangles (Qureshi *et al*., 2013; Jia *et al*., 2014), and mutant huntingtin (86Q) in an HD model (Jia *et al*., 2014), and they have been implicated in aggresome formation (Jia *et al*., 2014). Mu‐class GST variants are risk factors for PD (Wahner *et al*., 2007) as well as AD (de Mendonca *et al*., 2014). Heat‐shock protein defects have been implicated in PD (Yang *et al*., 2015) and AD (Ou *et al*., 2014), and HspB1 mutations are associated with familial motor neuron diseases (Muranova *et al*., 2015). Hsp90 and its co‐chaperones regulate tau and Aβ processing (Blair *et al*., 2014), and Hsp90 may specifically protect TDP‐43 from ROS‐induced aggregation (Chang *et al*., 2013a). The mitochondrial HSP70, also called ‘stress‐70’ or ‘mortalin’, is implicated in PD and *in vitro* longevity (Wadhwa *et al*., 2015). Lamin A mutants induce nuclear protein aggregation (Hubner *et al*., 2006). Laminins complex with Aβ, preventing its fibril formation (Henriques *et al*., 2014). Band 4.1‐like protein 1 binds and stabilizes dopamine receptors (Binda *et al*., 2002).

Tau is hyperphosphorylated in AD (6–12 sites/molecule, vs. 2–4 normally) and consequently forms oligomers with normal tau, which cohere to MAPs 1A, 1B, and 2 (Iqbal *et al*., 2013). Neurofilament light chain is a CSF biomarker of late‐onset AD (Skillback *et al*., 2014) and PD (Herbert *et al*., 2015). Peptidyl‐prolyl cis/trans isomerase A regulates protein folding at proline residues and affects tau aggregation (Blair *et al*., 2015). Peroxiredoxins 1 and 2 protect against huntingtin neurotoxicity (Pitts *et al*., 2012) and dopaminergic neurodegeneration (Hu *et al*., 2011). Phosphoglycerate mutase activity in brain decreases with aging and especially with AD (Meier‐Ruge *et al*., 1984), perhaps explained by its AD‐specific accumulation in aggregates. Secernin‐1 was identified as an early marker of neurodegeneration in a transgenic mouse overexpressing human tau (Chang *et al*., 2013b). Natural variation in sequestosome‐1, which escorts aggregates to autophagosomes, is associated with early‐onset AD (Cuyvers *et al*., 2015). Spectrin has been utilized as a model of spontaneous amyloid formation (Castello *et al*., 2015), and the spectrin β4 locus is one of three genes strongly hypermethylated in AD (Sanchez‐Mut *et al*., 2013). Phosphorylation of synapsin‐1 triggers neurotransmitter release but is blocked by binding huntingtin protein in HD (Xu *et al*., 2013). Syntaxin‐1 is a synaptic SNARE protein that forms age‐ and PD‐dependent co‐aggregates with α‐synuclein (Garcia‐Reitbock *et al*., 2010). Tenascin‐R co‐aggregates with APP at nodes of Ranvier on myelinated axons (Xu *et al*., 2014). Knockdown of UBA1 or talin‐2 increased aggregation of a polyglutamine array in *C. elegans* and of transgenic huntingtin (Htt‐Q74‐GFP) in cultured human HEK293 cells (Teuling *et al*., 2011). Frame‐shifted misreading of ubiquitin‐B inhibits proteasomes in transgenic mice and phenotypically mimics AD (Irmler *et al*., 2012). Vinculin is one of three extracellular matrix protein hubs in a protein‐interaction network of ‘age‐related disease proteins’ (Wolfson *et al*., 2009). Ubiquitin hybrid (fusion) genes, Uba52 and Uba80, encode ubiquitin fused to ribosomal proteins and are upregulated during tumor‐cell apoptosis (Han *et al*., 2012).

Surprisingly, pyruvate kinase suppresses Aβ fibril formation *in vitro*, as effectively as any known chaperone (Luo *et al*., 2014). Mitochondrial aconitase activity is reduced almost twofold in lymphocytes from AD relative to normal controls (Mangialasche *et al*., 2015). ATP synthase α is found on the cell surface of neurons, where it binds oligomeric Aβ to initiate neurotoxicity (Xing *et al*., 2013). Adenylate kinase overexpression is a common feature of AD, PD, ALS, and epilepsy (Boison & Aronica, 2015). Phosphatidylethanolamine‐binding protein is ~20% more abundant in hippocampus of AD than NC, where it is thought to inhibit proteasomes (Chen *et al*., 2006). Microglial γ‐enolase is neuroprotective in a mouse model of AD (Hafner *et al*., 2013). Astrocyte protein PEA‐15 protects neuronal cells *in vitro* against MPTP‐induced dopaminergic cell death (a model of PD) and raises dopamine levels in mouse striatum *in vivo* (Ahn *et al*., 2014). Aspartate aminotransferase and aldolase are major targets for deglycation repair by DJ‐1, which is highly induced in many cases of PD (Richarme *et al*., 2015). Neurofilament medium polypeptide is one of three plasma proteins strongly associated with ALS (Haggmark *et al*., 2014). Cytochrome c oxidase mutations and polymorphisms are associated with multiple neurodegenerative diseases, including AD (Loera‐Castaneda *et al*., 2014).

## Conclusions

Aggregates are characteristic of most or all neurodegenerative diseases and confirmed diagnosis typically depends on immunohistochemical detection of just one or a few of the best known proteins in foci typical of each pathology. However, it has become increasingly clear that there are many more proteins coalesced in aggregates, than have been identified (let alone pursued) to date. Moreover, the specific catalogue of aggregated proteins in each disease is far from random, and based on evidence presented here, probably reflects an ordered process of protein accrual. Similar aggregates form in nematode models of human neurodegenerative diseases, expressing a human transgene to produce a protein thought to initiate or ‘seed’ aggregation. These models can provide insights into the underlying processes that predispose to, and contribute to, aggregation. Further, they provide a facile platform on which to test the functional roles of each protein component.

In the current study, we have learned from this approach that Alzheimer disease aggregates, purified by affinity to antibodies against total tau or Aβ_1–42_/APP, are distinguished by several proteins that are specific to one type of aggregate, but nevertheless have in common the great majority of contributing proteins. PTMs are markedly increased in tau‐ and Aβ‐IP (but not total) aggregates when they were derived from AD, consistent with greater recruitment of modified proteins into AD‐specific aggregates, although not into other aggregates. Through a better understanding of the sequences of events that are shared by diverse aggregates, we may learn features that will constitute generic targets for small‐molecule interventions. At the same time, disease‐specific features may unveil nuances by which different seed proteins provoke aggregation and neurotoxicity in affected areas.

A remarkable conclusion from the current data is that aggregates, especially those specific to AD brain, contain many proteins that are far more abundant in AD than in control aggregates—*the majority of which play functional roles in the accrual process, and/or were previously implicated in neurodegenerative diseases and other pathologies*. This is such a frequent outcome that it is difficult to explain how so many candidate proteins could contribute mechanistically to a single disease. Many of these proteins were also reported to play roles in aging, but one feature they all share is the propensity for aggregation. We therefore propose that aggregation in general, and disease‐specific aggregates in particular, provide insights into proteostasis failure and its consequences, which in turn underlies age‐ and disease‐specific pathologies.

## Experimental procedures

### Proteomics methods

Tissues from caudal hippocampus, flash frozen and stored at −80 °C, were pulverized in a mortar and pestle cooled on dry ice as described (Ayyadevara *et al*., 2014). After a brief low‐speed spin (5 min at ~2200 g), protein was assayed (Bradford; Bio‐Rad) in supernatants and equal protein contents were pooled or analyzed individually as indicated. Samples were incubated with DYNAL Protein‐G magnetic beads coated with antibody against total tau (ab80579, Abcam) or Aβ_1–17_ (ab11132, Abcam). Bound aggregates rinsed 3×, were eluted, and brought to 0.1 m HEPES buffer with 1% v/v sarcosyl, 5‐mm EDTA, and protease inhibitors. After centrifugation 30 min at 100 000 g, pellets were resuspended in Laemmli buffer containing 2% w/v SDS and 0.5% v/v ß‐mercaptoethanol and heated 5 min at 95 °C. Proteins from each immuno‐pulldown (IP) and from total aggregates without IP (equal fractions based on *initial protein content* rather than IP recovery) were resolved on 1% SDS‐acrylamide gels, which were Coomassie stained, sliced, and excised for tryptic digestion *in situ* (Promega, Madison WI). Eluted peptides were analyzed by LC‐MS/MS as described (Byrum *et al*., 2013; Ayyadevara *et al*., 2014).

Proteins were identified by spectral match in the UniProtKB/SwissProt database. For tau‐IP2, electrophoresis was replaced by a nanoparticle‐adsorption protocol (Luchini *et al*., 2008). Details are provided as Data S1.

### Statistics

Significance statistics for protein raw counts (Table 1) were calculated using 2‐tailed chi‐squared tests with Yates’ correction; note that Fisher's exact test is inappropriate for 2 × 2 tables with large cell entries. For each protein, a 2 × 2 table contains AD and NC spectral counts in the top row, and the total for all proteins in the lower row, from which we assess significant enrichment in either AD or NC tissues. This is a very conservative approach, as it ‘corrects’ the biologically important observation that AD aggregates have more protein than NC aggregates. To determine the number of significant proteins expected by chance, we excluded proteins with insufficient counts in both cells to possibly attain significance. The number remaining (proteins adequately represented to achieve significance) was then multiplied by the alpha value, 0.05 (the threshold for nominal significance, without correction for multiple endpoints) to calculate the number expected by chance. In Table 1, the *minimal* count numbers required to achieve chi‐squared significance were 4, 10, 4, 10, 6, and 7, for spectral‐count columns numbered 1–6, respectively.

Significance statistics for Table 2 were calculated by unpaired, 2‐tailed *t*‐tests. As above, to calculate the expected numbers of nominally significant proteins (at *P *<* *0.05), an adjustment was made for proteins that were identified in too few samples to possibly attain significance. The cutoff was arbitrarily set at a minimum of 3 detections in *either* the AD or NC group, for each protein. That is, a protein was considered capable of achieving significance if it was identified in 3 or more individual samples in either the AD or NC group.

### Immunohistochemistry

Formalin‐fixed paraffin‐embedded hippocampal sections of human brain were cut in seven‐micrometer sections. The sections were heated in a 60 °C incubator for one hour, and then, paraffin was dissolved in xylene. Tissue was rehydrated through graded alcohol to water. Slides were immersed in citrate buffer (for antigen retrieval) as it cooled from boiling, over a span of 30 min. After blocking (60 min.) in serum‐free protein block (Dako, Carpinteria CA), slides were incubated overnight at 4 °C in a humidified container with one or more of the following antibodies: Dynactin/p50, 1:500 (Novus, Littleton CO; NBP2‐16115); AT8 (tau‐pSer202, pThr205), 1:100 (Thermo, Waltham MA; MN1020); LC3B, 1:50 (Abcam Cambridge, U.K.; ab65054); pan‐14‐3‐3, 1:50 (Santa Cruz Dallas TX; sc629); SQSTM1, 1:50 (Santa Cruz sc10117); tau‐pThr205, 1:50 (Santa Cruz sc101817); tau5, 1:50 (Abcam ab80579); Neurofilament, 1:1000 (Millipore, Billerica MA; AB5539); Alpha‐Internexin, 1:250 (Abcam ab40758), and MOAB‐2, 1:200 (kind gift from Leon Tai, Univ. of Illinois, Chicago).

Slides were incubated 1 h in the appropriate secondary antibodies (Invitrogen, Carlsbad CA): AF488 (A21202), AF594 (A21207), and AF647 (A21447 and A31573), followed by 2 min in 0.1% Sudan Black B in 70% ethanol to quench autofluorescence. Finally, DAPI (Thermo 62247), diluted 1:1000, was applied for 5 min to visualize nuclei. Slides were mounted under Prolong Gold medium, and the edges sealed with clear nail polish.

### Proximity ligation amplification

Sections used for Duolink PLA (Sigma‐Aldrich, St. Louis MO) were treated as IHC sections (see above) until 4 °C incubation with primary antibodies. After this step, slides were treated according to the manufacturer's protocol. In brief, slides were washed in successive PLA wash buffers, and incubated 1 h with oligonucleotide‐conjugated secondary antibodies, washed, and then treated with ligase, 30 min at 37 °C. Slides were again washed as above, covered in amplification solution containing polymerase and fluorescent probes, and incubated at 37 °C for 100 min. Slides were washed, blocked with 0.1% Sudan Black B in 70% ethanol to reduce autofluorescence, and mounted in DuoLink Mounting Medium with DAPI.

Immunofluorescence images were captured at identical exposure settings and with identical fluor‐specific filters, using a Nikon Eclipse E600 microscope (Melville, NY, USA) equipped with a Coolsnap ES monochrome camera (Photometrics, Tucson, AZ, USA). Images were taken through either 20× or 40× objective lenses, and reproduced at 120×, with the exception of Fig. 5E–F (100× objective, reproduced at 400×). Tissue samples for proteomic analyses were flash frozen in liquid nitrogen after dissection, placed on dry ice and stored at −80 °C. Formalin‐fixed paraffin‐embedded (FFPE) samples for immunohistochemistry were prepared in a standard histological processor.

## Funding

This work was supported by grants to RJSR (VA Merit, VA Senior Research Career Scientist Award), SA (subaward of NIH/NIA grant P30 AG028718 [J. Wei, P.I.]), SWB (NIH R03 AG043784 and P01 AG012411; Sturgis Charitable & Educational Trust); and WSTG (NIH P01AG012411; Windgate Charitable Foundation).

## Conflict of interests

The authors have no conflict of interests.

## Author contributions

SA and RJSR planned the proteomic experiments and functional assays in *C. elegans*; SA, RA, and MB performed all nematode studies and aggregate preparations; SWB and WSTG provided AD and NC hippocampal samples and thin sections, along with metadata; AJT, EP, and WZ performed proteomic analyses; PAP performed all immunohistochemistry and PLA assays; and RJSR wrote the paper with input from all authors.

## Supporting information


**Figure S1.** Enrichment of proteins and post‐translational modifications in pooled hippocampal tissue from AD relative to normal controls.

**Figure S2.** Hippocampal tau‐IP aggregate proteins, AD vs. AMC.

**Table S1.** Subject Data.

**Data S1**. Full proteomics data.
