# Supplementary figures and images for: Proteins that mediate protein aggregation and cytotoxicity distinguish Alzheimer's hippocampus from normal controls
Source: Aging Cell. 2016 Jul 23;15(5):924–39. doi: 10.1111/acel.12501 (PMC5013017; doi:10.1111/acel.12501)

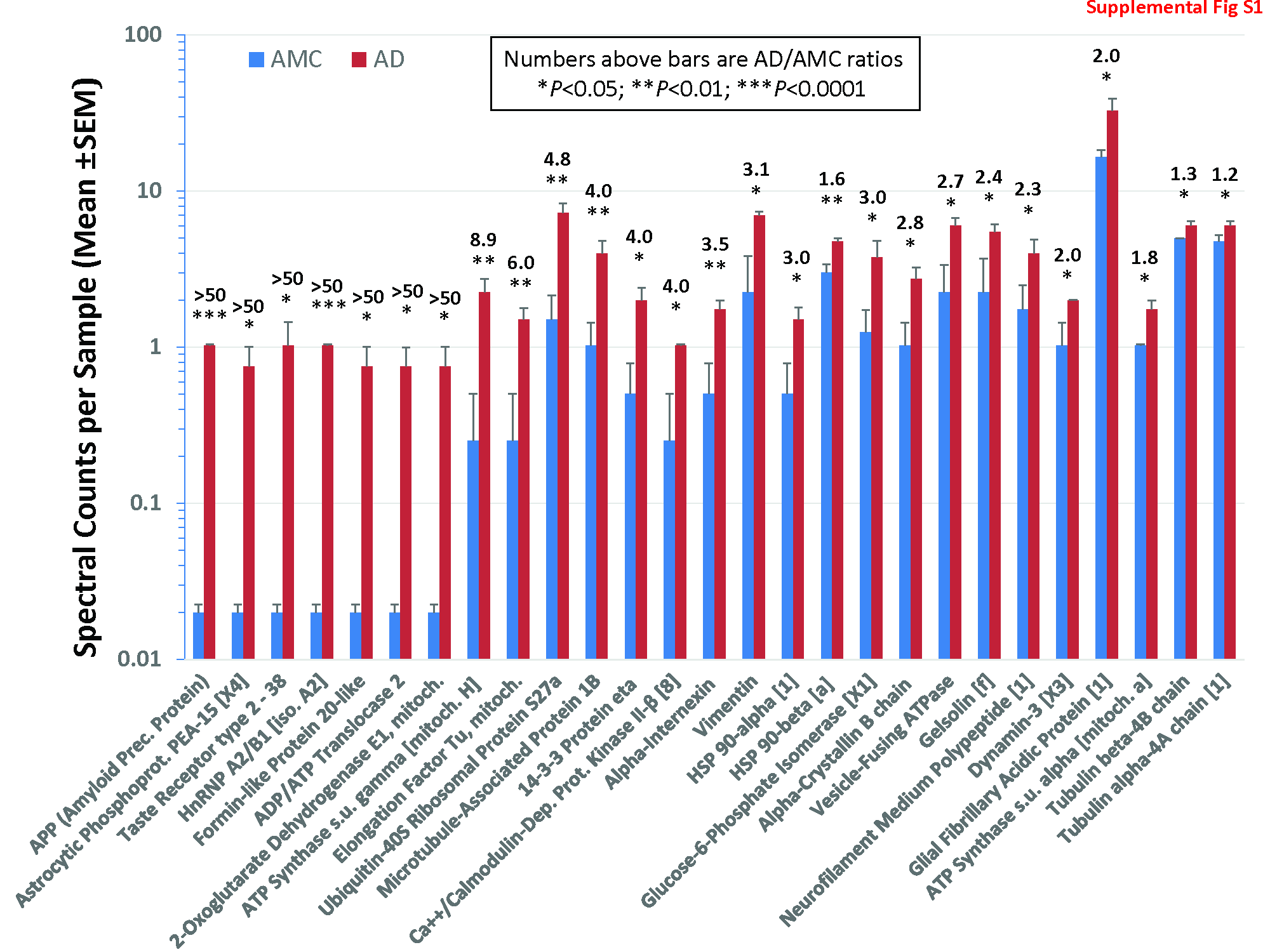

Supplement: Supplementary file 1 — Figure S1. Enrichment of proteins and post‐translational modifications in pooled hippocampal tissue from AD relative to normal controls. [file ACEL-15-924-s001.tiff]

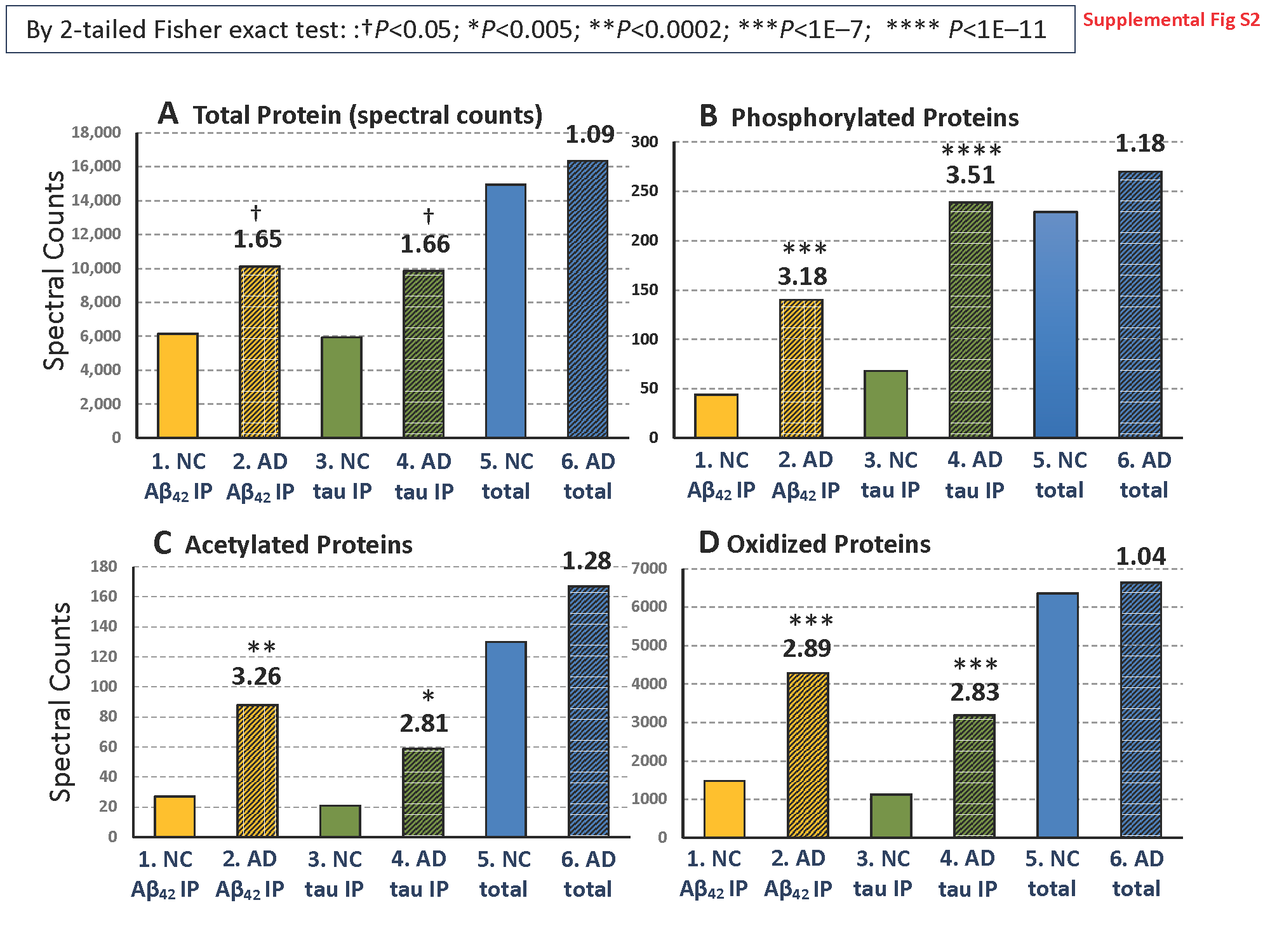

Supplement: Supplementary file 2 — Figure S2. Hippocampal tau‐IP aggregate proteins, AD vs. AMC. [file ACEL-15-924-s002.tiff]
