# Supplementary material for: Proteins that mediate protein aggregation and cytotoxicity distinguish Alzheimer's hippocampus from normal controls
Source: Aging Cell. 2016 Jul 23;15(5):924–39. doi: 10.1111/acel.12501 (PMC5013017; doi:10.1111/acel.12501)
Supplement: Supplementary file 3 — Table S1. Subject Data. [file ACEL-15-924-s003.docx]

**Figure S1. Enrichment of proteins and post-translational modifications in pooled hippocampal tissue from AD relative to normal controls.** Numbers over the AD bars indicate the frequency ratio of spectral hits in AD over control samples (AD/NC). Note that for the two IP fractions, these ratios are close to 2 (1.7–2.1) even after ‘normalization’ for the higher aggregate protein levels of AD samples.

**Figure S2. Hippocampal tau-IP aggregate proteins, AD vs. AMC**. Data correspond to ‘tau-IP 1’ columns of Table 2 (each *N* = 4), with the addition of several proteins omitted from Table 2 due to not meeting criteria for inclusion (≥1.5-fold change, supported in another analysis). Isoforms are indicated by brackets, e.g. [1]. Values of zero (protein not detected in any sample) have been replaced with 0.02 to allow plotting on the log-scale ordinate.

**Table S1. Subject Data.** A. Normal Control (NC) and AD Subjects for Tau-IP Comparison

| **Case #** | **Sample #** | **Diagnosis** | **B&B** | **ApoE** | **PMI** | **Age** | **Sex** | **Race** |
| --- | --- | --- | --- | --- | --- | --- | --- | --- |
| **04-005 (2018)** | **1** | **NC (CVD)** | **0** | **3,3** | **22** | **69** | **M** | **W** |
| **99-029** | **3** | **NC** | **0** | **3,3** | **9.5** | **71** | **M** | **W** |
| **05-064 (2048)** | **4** | **NC (CVD)** | **1** | **3,3** | **16** | **69** | **M** | **W** |
| **05-009 (2040)** | **9** | **NC (CVD)** | **1.5** | **3,3** | **17** | **69** | **M** | **W** |
| **Mean NC:** |  |  | **0.6** |  | **16.1** | **69.5** |  |  |
| **06-071 (1054)** | **6** | **AD** | **5** | **3,4** | **3** | **66** | **M** | **W** |
| **04-038 (UA 2028)** | **13** | **AD definite** | **3** | **3,3** | **19** | **85** | **M** | **W** |
| **VA07-11 (MRC 040249)** | **14** | **AD, severe** | **6** | **3,4** | **23** | **61** | **M** | **W** |
| **07-003 (MRC020031)** | **15** | **AD** | **5** | **3,4** | **4.5** | **77** | **M** | **W** |
| **Mean AD:** |  |  | **4.8** |  | **12.4** | **72.3** |  |  |
|  |  |  |  |  |  |  |  |  |

B. Additional Subjects for Multivariate Regression.

| **Case #** | **Sample #** | **Diagnosis** | **B&B** | **ApoE** | **PMI** | **Age** | **Sex** | **Race** |
| --- | --- | --- | --- | --- | --- | --- | --- | --- |
| **04-041 (2027)** | **2** | **NC** | **0** | **3,3** | **20** | **46** | **M** | **B** |
| **04-058 (2033)** | **5** | **NC/mild AD** | **4** | **3,4** | **14** | **70** | **M** | **W** |
| **05-017 (2042)** | **10** | **NC** | **0** | **3,3** | **13** | **56** | **M** | **W** |
| **98-122** | **7** | **AD** | **4.5** | **3,3** | **4** | **92** | **F** | **W** |
| **95-096** | **8** | **AD, severe** | **5** | **3,4** | **2** | **87** | **F** | **W** |
| **07-071 (1072)** | **11** | **AD, definite** | **4** | **4,4** | **12** | **86** | **F** | **W** |
| **04-050 (1030)** | **12** | **AD, definite** | **5** | **3,4** | **9.5** | **90** | **F** | **W** |

NC, normal control; AD, Alzheimer’s disease; B&B, staging of Alzheimer’s Disease based on criteria of Braak and Braak (as revised in (Braak et al., 2006)); ApoE, allele pair based on DNA genotype; PMI, postmortem interval.
