# Supplementary material for: Proteins that mediate protein aggregation and cytotoxicity distinguish Alzheimer's hippocampus from normal controls
Source: Aging Cell. 2016 Jul 23;15(5):924–39. doi: 10.1111/acel.12501 (PMC5013017; doi:10.1111/acel.12501)
Supplement: Supplementary file 4 — Data S1. Full proteomics data. [file ACEL-15-924-s004.docx]

**Detailed proteomics methods for Table 1 (pooled samples).**

Tissues from caudal hippocampus, flash frozen and stored at -80^o^C, were pulverized in a mortar and pestle cooled on dry ice as described [1]. After a brief low-speed spin (5 min at ~2200 g), supernatant protein concentrations were assayed (Bradford; Bio-Rad, Hercules CA). Equal amounts by protein content were pooled from 3 AD or 3 AMC individuals and incubated with monoclonal antibody against either total tau (ab80579, Abcam) or Aβ_1–17_ (ab11132, Abcam) attached to DYNAL protein-G magnetic beads. Bound aggregates were rinsed 3 times, eluted, and suspended in 0.1 M HEPES buffer with 1% v/v sarcosyl, 5-mM EDTA, and protease inhibitors. After centrifugation 30 min at 100 000 g, each pellet was resuspended in Laemmli buffer containing 2% w/v SDS and 0.5% v/v ß-mercaptoethanol and heated 5 min at 95°C. Proteins from each immuno-pulldown (IP) and from total aggregates without IP (equal fractions based on *initial protein content* rather than IP recovery) were resolved on 1% SDS-acrylamide gels and stained with Coomassie blue. Gel slices (including the well bottom to capture any undissolved aggregates) were robotically excised and proteins were digested *in situ* with trypsin (Promega). Peptides were eluted and analyzed as previously described [1], by high‐resolution LC‐MS/MS with a ThermoVelos Orbitrap mass spectrometer coupled to a Waters nanoACQUITY liquid chromatography system.

Proteins were identified by searching the UniProtKB SwissProt database (2013_12 release; restricted to *Homo sapiens*; 20,274 entries) using an in-house Mascot Sever (v 2.4.1; Matrix Science). Peak lists were generated from raw data files by MSFileReader (v2.2; Thermo Scientific) and ExtractMSn (January 2011 release; Thermo Scientific). Charge state deconvolution and deisotoping were not performed. No contaminants were excluded from the data set. Mascot search parameters were specified as follows: trypsin digestion with up to two missed cleavages; fixed carbamidomethyl modification of cysteine; variable oxidation of methionine; variable N-terminal acetylation; 2.0 ppm precursor ion tolerance; 0.50 Da fragment ion tolerance. The following oxidations were added as variable modifications where indicated: The following post-translational modifications were added as variable modifications where indicated: phosphorylation of serine, threonine, or tyrosine; acetylation of lysine; oxidation (+16) of histidine, tryptophan, or tyrosine (oxidation of methionine is included by default); sulfonation of methionine (+32); oxidation of tryptophan to kynurenine (+4), formylkinurenine (+32), or hydroxylformylkinurenine (+48). The false discovery rate (FDR) was estimated by a reverse-sequence decoy search [2].

**Detailed proteomics methods for Table 2, Tau-IP 2 (individual samples).**

Aggregate proteins were prepared from individual autopsy samples as described above for pooled samples, except that acrylamide gel separation was omitted. That is, individual samples were fractionated, aggregates isolated, and constituent proteins were directly analyzed by LC-MS/MS without prior electrophoretic separation. Proteins were identified by searching the UniProtKB SwissProt database (2014_05 release; restricted to *Homo sapiens*; 20,266 entries) using an in-house Mascot Sever (v 2.4.1; Matrix Science). Peak lists were generated from raw data files by MSFileReader (v2.2; Thermo Scientific) and ExtractMSn (January 2011 release; Thermo Scientific). Charge state deconvolution and deisotoping were not performed. No contaminants were excluded from the data set. Mascot search parameters were specified as follows: trypsin digestion with up to two missed cleavages; fixed carbamidomethyl modification of cysteine; variable oxidation of methionine; variable N-terminal acetylation; 2.0 ppm precursor ion tolerance; 0.50 Da fragment ion tolerance. The false discovery rate (FDR) was estimated by a reverse-sequence decoy search [2].

**Detailed proteomics methods for Table 2, Tau-IP 1 (individual samples).**

*Synthesis and Characterization of Hydrogel NIPAm/AAc Core-Shell Nanoparticles*

Particles were synthesized using NIPAm (Sigma-Aldrich) and BIS (Sigma-Aldrich) by precip­itation polymerization. Acrylic acid (AAc; Sigma-Aldrich) was incorporated into NIPAm particles to provide a charge-based affinity bait for capture of peptides and small molecules [3].

*Protein Capture and Isolation*

Aggregate proteins were prepared from individual autopsy samples as described above for Table 2, Tau-IP 2, without gel electrophoresis. Samples were centrifuged (7 min, 4°C, 16,000 rcf) and diluted 1:2 with 50 mM Tris-HCl, pH 7.0. Aliquots (0.4 ml) were spiked with proteins of known MW as internal process-control standards; nanoparticles were then added, incubating each sample 15 min at room temperature with slow rotation. After incubation for protein capture, samples were centrifuged (15 min, 25°C, 16,000 rcf), supernatants were discarded and particles were washed twice by resuspension in 1 mL of washing buffer (20% acetonitrile, 0.5×PBS) and centrifugation (4 min, 25°C, 16,000 rcf). Each pellet of washed particles was vortexed with 100 μL of elution buffer (60% acetonitrile/2% acetic acid) at room temperature. The elution mix was sonicated three times for 10 s each. The eluates from 3 successive elution steps were pooled, then lyophilized until dry, and stored at −20°C until use. Eluted proteins from the nanoparticles were reduced by dithiothreitol, alkylated by iodoacetamide, and digested with trypsin overnight at 37°C [3]. Each sample was taken from storage and dissolved just before loading and initiating each individual run. We note that similar high-quality MS result have been obtained from both the first and last sample, and quality-control parameters appear independent of loading order.

Peptides were analyzed by liquid chromatography-coupled tandem mass spectrometry (LC-MS/MS) using an LTQ-Orbitrap mass spectrometer (Thermo Fisher Scientific; Waltham, MA, USA) in data-dependent mode wherein each full MS scan (60,000 points) was followed by 8 MS/MS scans in which the 8 most abundant molecular ions at 1–3+ charge were fragmented by collision-induced dissociation (CID) [4]. The reversed-phase LC column, 100 µm × 10 cm, was slurry-packed with 5-μm, 200-Å pore size C_18_ resin (Michrom BioResources, Auburn, CA). Tandem mass spectra collected by Xcalibur (version 2.0.2) were searched against the NCBI human-protein database (2/2014 release, 71338 entries) using SEQUEST v. 3.3.1 (Bioworks software, ThermoFisher) with full tryptic-cleavage constraints, static iodoacetamide alkylation of cysteine, and variable methionine oxidation. Mass tolerance was 5 ppm for precursor ions and 0.25 Da for fragment ions. SEQUEST proteomics search results were filtered by criteria “Xcorr versus charge 1.8, 2.2, 3.0 for 1+, 2+, 3+ ions; ΔCn > 0.1; ranked top #1; P(random) < 0.01” and exported to Excel. The false discovery rate (FDR) was estimated by searching a combined forward-reverse database [2].

*References cited*

1. Ayyadevara S, Balasubramaniam MS, Gao Y, Yu L-R, Zybaylov B, Alla R, Shmookler Reis RJ. Proteins in aggregates functionally impact multiple neurodegenerative disease models by forming proteasome-blocking complexes. *Aging Cell* **2015**; 14:35–48. PMCID: PMC4326912.

# Elias JE, Gygi SP. Target-decoy search strategy for increased confidence in large-scale protein identifications by mass spectrometry. *Nat Methods* 2007; 4:207–214.

1. Luchini A, Geho DH, Bishop B, Tran D, Xia C, Dufour RL, Jones CD, Espina V, Patanarut A, Zhou WD, Ross MM, Tessitore A, Petricoin EF 3rd, Liotta L. Smart hydrogel particles: biomarker harvesting: one-step affinity purification, size exclusion, and protection against degradation. *Nano Lett* **2008**; 8:350–361.
2. Zhou W, Capello M, Fredolini C, Racanicchi L, Piemonti L, Liotta LA, Novelli F, Petricoin EF. Proteomic analysis reveals Warburg effect and anomalous metabolism of glutamine in pancreatic cancer cells. *J Proteome Res* **2012**; 11:554−563.
